# Cognitive decline in Dutch‐type hereditary and sporadic cerebral amyloid angiopathy: a 5‐year follow‐up study

**DOI:** 10.1002/alz.71629

**Published:** 2026-06-26

**Authors:** Rosemarie van Dort, Vera C. J. van Stek‐Smits, Sanne E. Schriemer, Reinier G. J. van der Zwet, Manon R. Schipper, Sabine Voigt, Ellen P. Hart, Vandhana Easwaran, Hamid R. Sohrabi, Kevin Taddei, Samantha L. Gardener, Ralph N. Martins, Steven M. Greenberg, Matthias J. P. van Osch, Marianne A. A van Walderveen, Marieke J. H. Wermer, Ellis S. van Etten

**Affiliations:** ^1^ Department of Neurology Leiden University Medical Center Leiden The Netherlands; ^2^ Department of Radiology Leiden University Medical Center Leiden The Netherlands; ^3^ Directorate of Research and Valorisation Leiden University Medical Center Leiden The Netherlands; ^4^ School of Psychology College of Health and Education Murdoch University Perth Western Australia Australia; ^5^ Centre for Healthy Ageing Health Futures Institute Murdoch University Perth Western Australia Australia; ^6^ Alzheimer's Research Australia Perth Western Australia Australia; ^7^ School of Medical and Health Sciences Edith Cowan University Joondalup Western Australia Australia; ^8^ The University of Western Australia Medical School Nedlands Western Australia Australia; ^9^ Massachusetts General Hospital and Harvard Medical School Harvard University Boston Massachusetts USA; ^10^ Department of Neurology University Medical Center Groningen Groningen The Netherlands

**Keywords:** amyloid beta (Aβ) deposition, cerebral amyloid angiopathy, cerebral small‐vessel disease (CSVD), cognitive decline, cognitive impairment, Dutch‐type cerebral amyloid angiopathy (D‐CAA), hereditary CAA, intracerebral hemorrhage (ICH), neurodegeneration, sporadic cerebral amyloid angiopathy (sCAA), vascular cognitive impairment

## Abstract

**INTRODUCTION:**

Cerebral amyloid angiopathy (CAA) is associated with cognitive impairment, but its longitudinal course of cognitive decline remains unclear. We investigated domain‐specific cognitive trajectories in Dutch‐type hereditary (D‐CAA) and sporadic CAA (sCAA) to compare patterns and rates of decline.

**METHODS:**

We included 181 participants – 93 D‐CAA mutation carriers (59 without, 34 with prior intracerebral hemorrhage [ICH]) and 88 with sCAA (57 without, 31 with ICH) – who underwent annual neuropsychological assessment. Longitudinal change in global cognition, memory, processing speed, and executive function was analyzed using linear mixed models.

**RESULTS:**

Over 5 years, cognitive decline was subtle but measurable. Memory and processing speed declined in D‐CAA with prior ICH, whereas D‐CAA without ICH and sCAA remained relatively stable. Global cognition showed a modest but significant decline, while executive function remained stable.

**DISCUSSION:**

CAA‐related cognitive decline appears domain‐ and stage‐dependent, suggesting progressive small‐vessel injury rather than acute hemorrhage may drive deterioration.

## Highlights

- Cognitive decline in CAA is subtle but measurable over time, showing domain‐ and stage‐specific patterns rather than overall deterioration.
- D‐CAA mutation carriers with a history of ICH showed a decline in memory and processing speed, whereas cognitive function remained relatively stable in D‐CAA mutation carriers without ICH and in patients with sCAA.
- Executive dysfunction was present at baseline across all CAA groups and remained stable over 5 years, suggesting a stable, early‐onset deficit possibly related to small‐vessel pathology.

## 1 BACKGROUND

Cerebral amyloid angiopathy (CAA) is a cerebral small‐vessel disease (CSVD) characterized by the accumulation of amyloid beta (Aβ) protein in the walls of leptomeningeal and cortical blood vessels.^1^, ^2^ Over time, this process can lead to progressive vascular injury associated with macro‐ and microstructural brain tissue abnormalities, which may manifest as cognitive and neurological impairments.^3^ Cognitive impairment associated with CAA may represent an important public health concern in a rapidly aging population.^4^, ^5^ Notably, nearly one quarter of the general population shows moderate to severe CAA pathology on histopathological examination, highlighting its high prevalence in the aging population.^6^ Although CAA pathology in the general population is often considered largely asymptomatic, its full clinical impact remains unclear, and cognitive impairment may represent an important clinical manifestation in patients with clinically diagnosed CAA.^3^, ^7^ The impact of CAA on cognition is influenced by several factors, including the extent of amyloid deposition, the severity of associated vascular pathology, and the occurrence of clinical events, such as intracerebral hemorrhage (ICH).^3^, ^8^, ^9^ Moreover, concomitant Alzheimer's disease (AD) pathology represents a major complicating factor in research on CAA‐related cognitive impairment, as overlapping amyloid‐ and tau‐related pathology can obscure the independent cognitive effects of CAA.^10^

In recent decades, research has increasingly focused on the cognitive consequences of CAA, often drawing comparisons with AD.^11^, ^12^, ^13^ Although both conditions often overlap, likely related to the shared role of Aβ deposits,^14^ CAA is typically associated with more pronounced deficits in executive function and perceptual speed, whereas typical sporadic AD often presents with more prominent (episodic) memory impairment, though substantial heterogeneity exists across age and disease stage.^15^, ^16^, ^17^, ^18^, ^19^ Despite growing evidence on the cognitive effects of vascular amyloid, most studies are cross‐sectional, and the longitudinal course of cognitive changes in CAA remains poorly understood.

Dutch‐type hereditary CAA (D‐CAA) represents a “pure” form of CAA, as it is an autosomal‐dominant disease that occurs without age‐related comorbidities such as AD, which is characterized by both extracellular Aβ plaques and tau‐mediated neurofibrillary tangles. These parenchymal plaques and tangles are generally absent in D‐CAA but may be present in sporadic CAA (sCAA) due to its frequent overlap with Alzheimer‐type pathology.^3^ While D‐CAA has an earlier onset and faster progression, its pathological and clinical characteristics seem to be relatively similar to sCAA. Therefore, D‐CAA is a unique model to investigate the direct effects of vascular amyloid depositions on cognition, without the confounding influences of AD and other age‐related vascular risk factors. Our previous cross‐sectional study revealed early deficits in executive function and memory, even before the first symptomatic ICH (sICH), suggesting an independent effect of vascular pathology on cognitive dysfunction in (D‐)CAA.^20^ These findings underscore the need for longitudinal studies to clarify the timing and progression of cognitive decline in CAA. Therefore, we aimed to characterize longitudinal domain‐specific cognitive trajectories in both D‐CAA and sCAA.

## 2 METHODS

### 2.1 Participants and study design

**RESEARCH IN CONTEXT**

1. **Systematic review**: The authors reviewed the literature using traditional (e.g., PubMed) sources. Previous reports on CAA, including D‐CAA and sCAA, have primarily used cross‐sectional designs to study cognition. While CAA is increasingly recognized as a contributor to cognitive impairment, longitudinal data on domain‐specific cognitive trajectories in CAA remain scarce.
2. **Interpretation**: Our findings show that cognitive decline in CAA is modest but detectable over time and follows a domain‐ and stage‐specific pattern. Progressive declines in memory and processing speed were observed primarily in patients with symptomatic D‐CAA (ICH+), whereas patients with presymptomatic D‐CAA (ICH−) and patients with sCAA (ICH− and ICH+) remained largely stable. Executive dysfunction was already present across all CAA groups at baseline while not showing further decline. These results suggest that vascular amyloid pathology alone can drive subtle but progressive cognitive impairment, particularly when symptomatic disease stages are reached, as progression occurred only in patients with prior ICH and not after new hemorrhagic events, indicating an underlying small‐vessel disease process rather than post‐ICH effects.
3. **Future directions**: Future studies should extend the follow‐up period and include biomarker data to disentangle CAA from co‐pathologies. Given the subtle cognitive changes observed over limited follow‐up, cognition may be best included as a secondary or exploratory outcome in future CAA treatment trials rather than as a primary endpoint.

This prospective, longitudinal cohort study included participants with D‐CAA and sCAA who participated in our natural history studies on disease progression and biomarkers in D‐CAA (AURORA study and its substudy TRACK D‐CAA) and sCAA (FOCAS‐study) between February 14, 2018, and January 27, 2024, in the Leiden University Medical Center (LUMC). Inclusion criteria for the D‐CAA mutation carriers were age ≥ 18 years and a genetically proven amyloid precursor protein (APP) mutation. D‐CAA was divided into groups based on a history (D‐CAA ICH+) or absence of (D‐CAA ICH−) sICH. sICH was specified as a symptomatic ICH confirmed on computed tomography (CT) or magnetic resonance imaging (MRI).

Patients with sCAA were all diagnosed as “probable CAA” according to the Boston criteria 2.0 and had no family history suggestive of hereditary CAA.^21^ They presented with lobar sICH, subjective cognitive decline, or transient focal neurological episodes (TFNEs). A minority initially presented with other reasons (e.g., ischemic stroke) but were found to meet radiological criteria for probable CAA during workup. Concomitant AD pathology was not formally excluded with additional diagnostics (e.g., amyloid positron emission tomography [PET] or cerebrospinal fluid). In the main analyses, the sCAA group was not subdivided by ICH status, as this would have created two heterogeneous subgroups differing in age and vascular comorbidity. Unlike D‐CAA, in which symptomatic ICH marks a well‐defined transition between presymptomatic and symptomatic stages due to its hereditary and predictable course, sCAA is typically diagnosed after clinical manifestations such as hemorrhage or cognitive decline, resulting in a more heterogeneous disease spectrum. Therefore, sCAA was analyzed as a single group in the main models, with stratification by ICH status performed only in a sensitivity analysis.

Approval for the studies was granted by the local Medical Ethics Committee Leiden Den Haag Delft (METC‐LDD). Written consent was obtained from all participants before their enrollment. The data from this study are available upon written request, including a proposal of the planned analyses and aims, and following completion of a data transfer agreement.

### 2.2 Data collection

Participants underwent a research day at baseline and after 1, 2, 3, and 5 years of follow‐up. During each research day, a neuropsychological assessment was conducted, and data on demographics, medical history, and clinical symptoms, including a history of sICH, were prospectively obtained. Information about prior sICH was obtained from electronic patient files. For descriptive purposes, MRI markers of CSVD were recorded at baseline according to the STandards for ReportIng Vascular changes on nEuroimaging (STRIVE) criteria, including white matter hyperintensities (WMHs, rated using the Fazekas scale), centrum semiovale enlarged perivascular spaces (EPVSs, grade ≥ 2, indicating multiple, clearly visible spaces), cortical superficial siderosis (cSS), and cerebral microbleeds (CMBs).^22^ These MRI characteristics were included to provide a comprehensive overview of disease burden but were not analyzed longitudinally.

### 2.3 Cognitive assessment

All neuropsychological tests were administered by trained researchers according to the testing manual. Participants annually received a standardized and comprehensive neuropsychological assessment, which consisted of the following validated instruments: Montreal Cognitive Assessment (MoCA) version 7.1,^23^ Trail Making Test Parts A and B (TMT‐A, TMT‐B),^24^ Rey Auditory Verbal Learning Test–15 words (RAVLT),^25^, ^26^ and Digit Span Test (forward and backward).^27^ Due to an amendment to the neuropsychological test battery in June 2019 (AURORA and FOCAS studies), a subgroup of 55 participants who completed their visit before this amendment underwent the test battery without the inclusion of the RAVLT. During a limited period following COVID‐19‐related interruptions in data collection, a large number of follow‐up visits needed to be rescheduled within a limited timeframe. To ensure that as many participants as possible could be reassessed, the protocol was temporarily adapted and only the MoCA was administered during these visits.

Global cognition was assessed with the MoCA (total score).^23^ The uncorrected MoCA total score was used in the analyses. Memory was evaluated by the RAVLT (total score on trials 1 to 5 and number correct on delayed recall and the number of correctly recognized target words on the recognition trial),^25^, ^26^ processing speed was quantified using TMT‐A (time in seconds), and executive function was measured through the TMT B/A ratio (as a measure for cognitive flexibility),^28^ and the Digit Span Test (longest span correct).^27^ The grouping of individual tests into domain‐specific composite scores was based on established neuropsychological constructs and defined before statistical analyses to capture broader cognitive functions. Years of education and education level were retrieved and classified, ranging from 1 (low) to 7 (high). Level of education was categorized as the highest completed level (1 to 3 low, 4 to 5 middle, 6 to 7 high level): 1: primary education; 2: lower vocational education; 3: low‐level secondary education or ≤3 years mid‐ to high‐level secondary education; 4: secondary vocational education; 5: average‐ or high‐level secondary education; 6: university of applied sciences; 7: university degree.^29^ By design, during annual follow‐up, neuropsychological testing was performed without blinding to information gathered at baseline.

### 2.4 Statistical analyses

Data are presented for D‐CAA ICH−, D‐CAA ICH+, and sCAA. Baseline group differences between the three CAA groups (D‐CAA ICH−, D‐CAA ICH+, and sCAA) were evaluated using independent‐samples *t*‐tests or Mann–Whitney U tests for continuous variables and chi‐squared (χ^2^) tests for categorical variables, as appropriate. Results are presented as means with standard deviations (SD) or medians with interquartile ranges (IQR), as appropriate. For descriptive analyses of follow‐up completeness (Table S1), we summarized the total number of neuropsychological assessments available per group at each year of follow‐up. These counts represent visits rather than unique individuals. Missing outcome data resulted from several sources, including protocol amendments, COVID‐19‐related restrictions during which only the MoCA was administered, incomplete follow‐up visits, and early termination of neuropsychological assessments. An overview of outcome availability across cognitive domains is provided in Table S2. For the global cognition, memory, processing speed, and executive function domains, raw test scores were entered into the Advanced Neuropsychological Diagnostics Infrastructure (ANDI) normative database to obtain age‐, sex‐, and education‐adjusted *Z*‐scores based on Dutch population norms.^26^, ^30^ These *Z*‐scores were then averaged into domain‐specific composite scores. Aborted tests were assigned a *Z*‐score of −3 SD to account for failed attempts. The *Z*‐scores for each cognitive domain were derived at each annual follow‐up visit. Because cognitive outcomes were expressed as standardized *Z*‐scores, longitudinal changes were interpreted relative to the normative distribution of the scores. To facilitate clinical interpretation, we examined the proportion of participants with domain‐specific cognitive dysfunction at baseline. Cognitive dysfunction was defined as *Z* ≤ −1 SD, with a more stringent cut‐off of Z ≤ −2 SD to indicate more pronounced impairment, in line with commonly used thresholds. To additionally explore clinically meaningful longitudinal change, we calculated the proportion of participants showing a decline of ≥1 SD from their baseline *Z*‐score in each cognitive domain during follow‐up.

Longitudinal change over the 5 years was analyzed using linear mixed‐effects models (LMMs) to account for repeated measurements within individuals and varying follow‐up durations. Separate models were constructed for each cognitive domain composite score derived from the individual cognitive tests, with time (years since baseline) as a continuous variable to assess longitudinal change. Fixed effects included time, CAA group (D‐CAA ICH−, D‐CAA ICH+, and sCAA), and their interaction (Time × Group) to assess whether the rate of cognitive change differed between groups, as well as visit‐specific sICH status (yes/no), a time‐varying covariate indicating whether the assessment occurred within a year after sICH. Cognitive test scores were already adjusted for age, sex, and education level using standardized normative data (ANDI database). This approach allows for comparability across domains while avoiding redundancy from re‐entering these covariates in the mixed‐effects models. All models included a random intercept for participant ID to account for within‐subject correlation due to repeated measurements over time. All group effects were estimated with sCAA as the reference category, enabling direct comparison between hereditary (D‐CAA ICH− and D‐CAA ICH+) and sporadic CAA. This reference choice facilitated interpretation of *β* coefficients as relative differences in both cognitive level and rate of change compared with the clinically typical sporadic form. All tests were two‐sided (*α* = 0.05). All analyses were performed in R version 4.3.2 (R Foundation for Statistical Computing, Vienna, Austria). Linear mixed‐effects models were fitted using the *lme4* and *lmerTest* packages, and estimated marginal means were computed with *emmeans*. Data management and visualization were performed using the *tidyverse* suite (including *dplyr*, *ggplot2*, and *tidyr*).

#### 2.4.1 Sensitivity analyses

Two sets of sensitivity analyses were performed. First, to assess whether short‐term effects of sICH influenced cognitive trajectories, we re‐estimated all models after excluding follow‐up assessments conducted within a year after a sICH (Section 3.4 and Table S3).

Second, we examined whether ICH status influenced the longitudinal results in sCAA. Although the sCAA subgroups (ICH− and ICH+) are relatively heterogeneous, this subdivision was performed as a sensitivity analysis to evaluate whether the absence of stratification in the main models affected the results. Therefore, the sCAA group was subdivided into patients without (sCAA ICH−) and with (sCAA ICH+) prior sICH, and all mixed‐effects models were repeated using the same model specifications (Section 3.5, Table S4, S5, and Figure S1).

## 3 RESULTS

### 3.1 Study population

**FIGURE 1:**
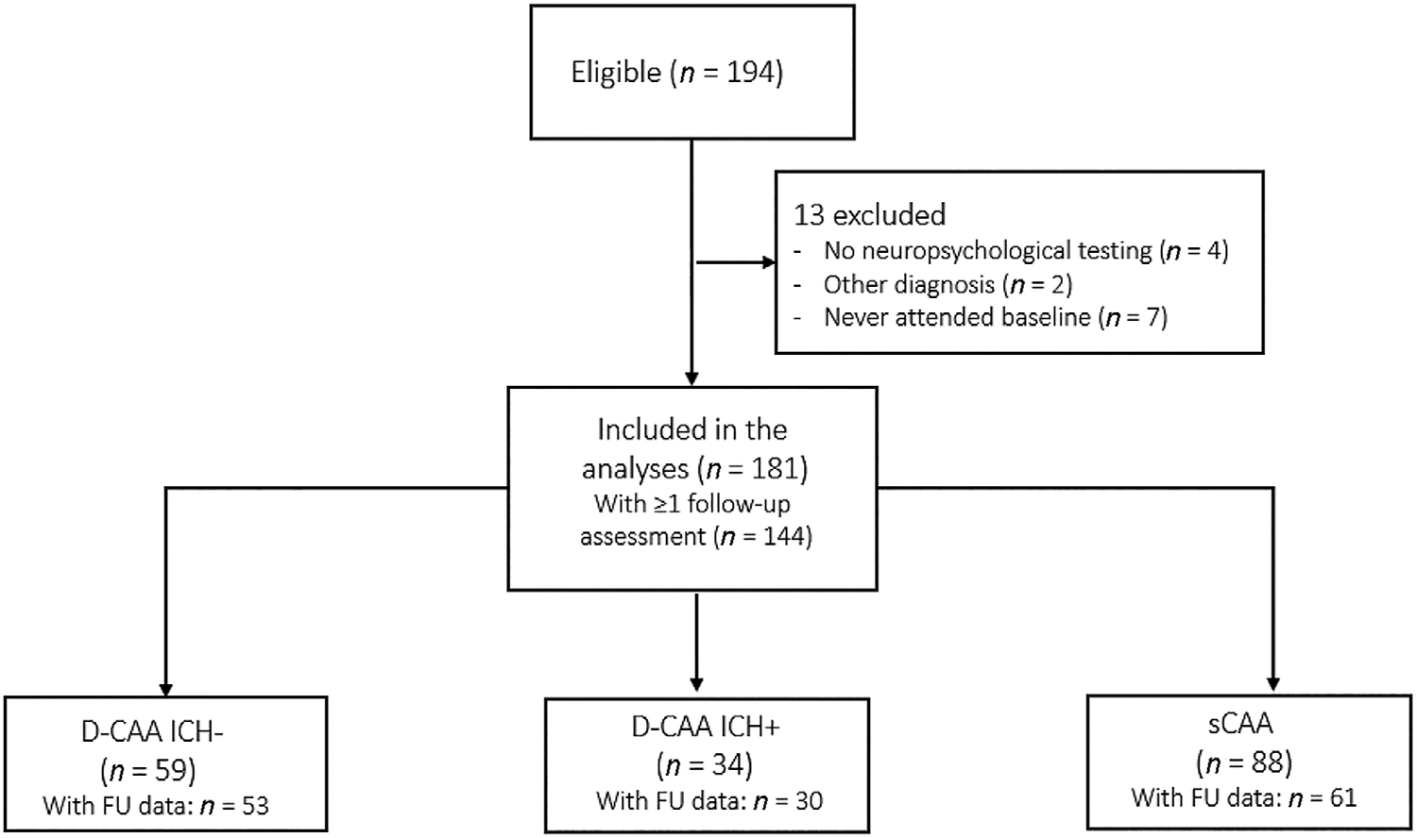
Flowchart inclusions.

A total of 181 participants were included: 93 D‐CAA mutation carriers (59 ICH−, 34 ICH+) and 88 patients with sCAA (57 ICH−, 31 ICH+) (Table 1). Patients with sCAA were older (mean 70.2 ± 6.5 years) than D‐CAA ICH+ (57.7 ± 7.3) and D‐CAA ICH− (40.2 ± 10.3). At baseline, the proportion of sICH relative to the total number of macrobleeds was 31.2% in D‐CAA ICH+ and 44.4% in sCAA. Follow‐up neuropsychological data were available for 53 D‐CAA ICH− (89.8%), 30 D‐CAA ICH+ (88.2%), and 61 sCAA (69.3%) participants. Completeness of follow‐up varied, with 34% of D‐CAA ICH−, 71% of D‐CAA ICH+, and 28% of sCAA participants completing three or more annual assessments, while only 10% to 12% had baseline data only (Figure 1, Table S1). Availability of cognitive outcome data differed across cognitive domains, with the highest missingness observed for memory and executive function (Table S2).

**TABLE 1:** Baseline characteristics.

Characteristic	D‐CAA ICH‐	D‐CAA ICH+	sCAA
*N*	59	34	88
Age, mean, years (SD)	40.2 (10.3)	57.7 (7.3)	70.2 (6.5)
Female, *n* (%)	35 (59.3)	15 (44.1)	36 (40.9)
First CAA‐related complaints, *n* (%)			
ICH	0 (0)	22 (11.8)	29 (33.0)
(Subjective) cognitive decline	5 (8.5)	4 (5.9)	5 (5.7)
Transient focal neurological episodes	0 (0)	1 (2.9)	11 (12.5)
Seizures	1 (1.7)	4 (11.8)	0 (0)
Convexity subarachnoid hemorrhage	0 (0)	0 (0)	3 (3.4)
Other	1 (1.7)	1 (2.9)	12 (13.6)
None/genetic testing/research	44 (74.6)	0 (0)	0 (0)
History of symptomatic ICH, *n* (%)*	–	34 (100)	31 (35.2)
Number of symptomatic ICH, median [IQR]	–	2 [1 to 3]	1 [0–1]
First ICH location, *n* (%)			
Frontal	–	8 (23.5)	13 (38.2)
Parietal	–	11 (32.4)	8 (23.5)
Temporal	–	8 (23.5)	6 (17.6)
Occipital	–	7 (20.6)	6 (17.6)
Cerebellar	–	0 (0)	1 (2.9)
History of depression, *n* (%)	9 (15.5)	6 (17.6)	17 (19.5)
History of subjective personality change ^†^, *n* (%)	14 (23.7)	19 (55.9)	53 (60.9)
History of apathy, *n* (%)	6 (10.2)	7 (20.6)	14 (16.1)
Education, years (SD)	14.3 (2.7)	13.1 (3.4)	14.2 (3.9)
Education level, *n* (%)			
High	19 (32.8)	9 (30)	25 (36.2)
Average	28 (48.2)	9 (30)	22 (32.4)
Low	11 (19.0)	12 (40.0)	21 (30.8)
APOE ε4 carrier, *n* (%)	22 (38.6)	13 (61.9)	38 (53.5)
Macrobleeds, median [IQR]	0 [0–0]	5 [2‐11]	2 [0–2]
Cortical superficial siderosis, *n* (%)	7 (13)	22 (68.8)	44 (57.9)
Cerebral microbleeds, mean (SD)	32 (90)	315 (362)	144 (196)
CSO‐EPVS grade, median [IQR]	3 [2–4]	4 [4‐4]	4 [3–4]
Fazekas DWMH grade, median [IQR]	1 [0–1]	2 [2‐3]	2 [1–2]
Hypertension, *n* (%)	12 (20.3)	10 (29.4)	44 (51.2)
Hypercholesterolemia, *n* (%)	5 (8.6)	12 (36.4)	37 (45.1)
Diabetes mellitus type II, *n* (%)	0 (0)	3 (8.8)	7 (8.0)
Smoking (ever), *n* (%)	41 (69.5)	21 (61.8)	60 (69.0)
Alcohol use (ever), *n* (%)	54 (91.5)	28 (82.4)	76 (87.4)

Abbreviations: CSO‐EPVS, centrum semiovale enlarged perivascular spaces; D‐CAA, Dutch‐type cerebral amyloid angiopathy; DWMH, deep white matter hyperintensities; ICH, intracerebral hemorrhage; sCAA, sporadic cerebral amyloid angiopathy.

* Recorded only for patients with a history of symptomatic ICH.

^†^ Collected as personal experience of change in character in most recent years. Education level data were missing for one D‐CAA ICH−, four D‐CAA ICH+, and 20 sCAA participants; percentages are based on available data. APOE data were available for 57 D‐CAA ICH−, 21 D‐CAA ICH+, and 71 sCAA participants; percentages are based on available data.

### 3.2 Baseline cognitive performance

At baseline, participants with D‐CAA ICH− performed within the normal range for global cognition (MoCA mean *Z* = 0.34 ± 0.9), processing speed (0.47 ± 1.1), and executive functioning (TMT‐B *Z* = 0.54 ± 1.2; cognitive flexibility *Z* = 0.62 ± 1.3) but showed slightly lower performance in memory compared with other cognitive domains, while scores remained within normative reference ranges (RAVLT immediate recall *Z* = −0.58 ± 1.6; delayed recall *Z* = −0.20 ± 1.4) (Table 2). Patients with D‐CAA ICH+ exhibited lower performance in memory (immediate recall *Z* = −0.58 ± 0.6; delayed recall *Z* = −0.02 ± 1.4), processing speed (TMT‐A *Z* = 0.04 ± 1.0), and executive functioning (TMT‐B *Z* = −0.11 ± 1.5) compared with D‐CAA ICH−. Patients with sCAA showed the lowest performance across domains, particularly in memory (immediate recall *Z* = −0.95 ± 0.8; delayed recall *Z* = −1.04 ± 1.1), processing speed (*Z* = −0.30 ± 1.5), and executive functioning (TMT‐B *Z* = −0.44 ± 1.6; all *p* < 0.05 vs D‐CAA ICH−).

**TABLE 2:** Cognitive function at baseline.

Cognitive domain	D‐CAA ICH−	D‐CAA ICH+	sCAA	*p*‐value	*p*‐value
*n (baseline)*	59	34	88	(ICH+ vs ICH−)	(sCAA vs. ICH‐)
Global cognition				0.616	<0.001
MoCA, median [IQR]	28 [26 to 29]	27 [24 to 28]	25 [23 to 27]		
MoCA, *Z*‐score mean (SD)	0.336 (0.9)	0.144 (1.1)	−0.423 (1.2)		
Memory				0.936	0.041
RAVLT immediate recall, median [IQR]	44 [37 to 49]	32 [28 to 45]	29 [24 to 38]		
RAVLT delayed recall, median [IQR]	9 [7 to 12]	6 [6 to 9]	5 [3 to 7]		
RAVLT recognition, median [IQR]	29 [28 to 30]	27 [27 to 29]	27 [23 to 28]		
Immediate recall, *Z*‐score mean (SD)	−0.575 (0.9)	−0.580 (1.6)	−0.951 (0.8)		
Delayed recall, *Z*‐score mean (SD)	−0.449 (1.1)	−0.200 (1.4)	−1.043 (1.1)		
Recognition, *Z*‐score mean (SD)	−0.578 (0.9)	−0.768 (0.6)	−0.772 (1.0)		
Processing speed				0.094	0.001
TMT‐A, seconds, median [IQR]	22 [19 to 30]	35 [27 to 43]	47 [30 to 59]		
TMT‐A, *Z*‐score mean (SD)	0.470 (1.1)	0.042 (1.0)	−0.302 (1.5)		
Executive function				0.656	0.634
TMT‐B, seconds, median [IQR]	51 [37 to 72]	73 [56 to 118]	95 [62 to 138]		
Cognitive flexibility (TMT B/A), mean (SD)	2.342 (0.7)	2.509 (1.0)	2.569 (1.0)		
TMT‐B, *Z*‐score mean (SD)	0.543 (1.2)	−0.105 (1.5)	−0.443 (1.6)		
Cognitive flexibility, *Z*‐score mean (SD)	0.623 (1.3)	0.582 (1.5)	0.680 (1.6)		
Digit Span forward, median [IQR]	6 [5 to 7]	6 [5 to 6]	5 [5 to 6]		
Digit Span backward, median [IQR]	4 [4 to 5]	4 [3 to 5]	4 [3 to 5]		
Digit Span forward, *Z*‐score mean (SD)	−0.982 (0.8)	−1.002 (0.9)	−1.170 (0.8)		
Digit Span backward, *Z*‐score mean (SD)	−2.674 (1.1)	−2.240 (1.3)	−2.429 (1.1)		

*Note*: Higher *Z*‐scores = better performance; for time‐based TMT measures, fewer seconds correspond to higher *Z*‐values. *P* values represent pairwise comparisons between CAA groups, with D‐CAA ICH− as the reference group. Baseline comparisons are descriptive and were not adjusted for multiple comparisons.

Abbreviations: B/A, ratio between completion times of Part B and A; IQR, interquartile range; MoCA, Montreal Cognitive Assessment; RAVLT, Rey Auditory Verbal Learning Test; SD, standard deviation; TMT‐A/B, Trail Making Test Parts A and B.

To facilitate clinical interpretation, we examined the proportion of participants with domain‐specific cognitive dysfunction at baseline (Z ≤ −1 SD). More severe dysfunction (Z ≤ −2 SD) was less common (Table S6).

### 3.3 Longitudinal cognitive decline

#### 3.3.1 Global cognition

**FIGURE 2:**
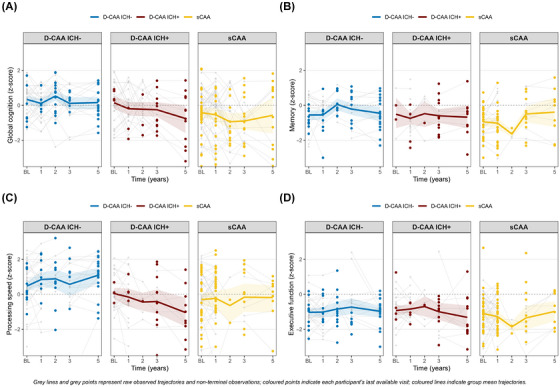
Cognitive trajectories.

Global cognition showed a modest but statistically significant decline over time in the overall CAA sample (*F*[1, 424.9] = 20.99, *p* < 0.001; *β* = −0.172 ± 0.048). Longitudinal cognitive trajectories across domains are illustrated in Figure 2, and the results of the LMMs are presented in Table 3. At each assessment, cognitive performance was standardized relative to age‐, sex‐, and education‐adjusted Dutch normative data derived from the ANDI database. However, as these norms are based on cross‐sectional data, no formal comparison with longitudinal age‐related cognitive decline was performed, and this finding should therefore be interpreted with caution.^30^ Baseline cognitive levels did not differ significantly between groups (*F*[2, 473.9] = 0.630, *p* = 0.533). Model‐based means (estimated marginal means, adjusted for baseline, group, Time × Group interaction, and visit‐specific sICH) suggested slightly higher overall performance in D‐CAA ICH− (0.055 ± 0.079) and lower scores in D‐CAA ICH+ (−0.183 ± 0.104) and sCAA (−0.352 ± 0.075). Group differences in slopes were modest: The decline in D‐CAA ICH− was significantly slower than in sCAA (Δ = 0.407 SD, 95% confidence interval [CI]: 0.141 to 0.673, *p* < 0.001), whereas D‐CAA ICH+ did not differ from either D‐CAA ICH− (Δ = 0.238 SD, *p* = 0.218) or sCAA (Δ = 0.169 SD, *p* = 0.581). Post hoc within‐group slopes showed that D‐CAA ICH+ exhibited a significant decline (*β* = −0.183 SD per visit, 95% CI: −0.294 to −0.072, *p* < 0.001 vs 0), sCAA showed a similar modest decline (*β* = −0.166, 95% CI: −0.258 to −0.074, *p* = 0.002 vs 0), and D‐CAA ICH− showed only a modest and non‐significant change (*β* = −0.042, 95% CI: −0.128 to 0.044, *p* = 0.33 vs 0). These findings indicate that although decline was present across CAA patients, only D‐CAA ICH+ showed a clearly steeper within‐group deterioration. The Time × Group interaction was borderline significant (*F*[2, 426.0] = 3.01, *p* = 0.050), consistent with modest stage‐dependent differences in trajectories. Neither a new sICH (*p* = 0.538) nor its interaction with time (*p* = 0.424) affected global cognition. As expected, baseline performance strongly predicted follow‐up scores (*β* = 0.728 ± 0.044, *F*[1, 203.0] = 274.8, *p* < 0.001).

**TABLE 3:** Linear mixed‐effects results.

Cognitive domain	Group contrast	*β* (adjusted)	95% CI	*p* value	Group slope	*p* value(vs sCAA)	sCAA mean	sCAA slope
Global cognition	(D‐CAA ICH−)—sCAA	0.403	0.157 to 0.649	0.001	−0.042 (−0.128 to +0.044)	0.103	−0.346	−0.166
Global cognition	(D‐CAA ICH+)—sCAA	0.169	−0.115 to 0.453	0.315	−0.183 (−0.294 to −0.072)	0.950	−0.346	−0.166
Memory	(D‐CAA ICH−)—sCAA	0.030	−0.174 to 0.235	0.909	+0.260 (+0.152 to +0.369)	0.790	−0.578	0.211
Memory	(D‐CAA ICH+)—sCAA	−0.111	−0.515 to 0.292	0.747	−0.130 (−0.362 to +0.102)	0.026	−0.578	0.211
Processing speed	(D‐CAA ICH−)—sCAA	0.224	−0.026 to 0.473	0.086	+0.147 (+0.063 to +0.231)	0.307	0.082	0.057
Processing speed	(D‐CAA ICH+)—sCAA	−0.275	−0.561 to 0.010	0.061	−0.204 (−0.313 to −0.094)	0.001	0.082	0.057
Executive function	(D‐CAA ICH−)—sCAA	0.072	−0.116 to 0.261	0.596	+0.036 (−0.036 to +0.108)	0.939	−1.083	0.021
Executive function	(D‐CAA ICH+)—sCAA	0.047	−0.175 to 0.270	0.834	−0.025 (−0.118 to +0.069)	0.699	−1.083	0.021

*Note*: sCAA served as the reference group. Group slope = adjusted change (SD per visit) for each group. Positive slope values indicate improvement over time, whereas negative values indicate decline. 95% confidence intervals and *p* values for *β* and slope represent Bonferroni‐adjusted pairwise contrasts versus sCAA. The final *p* value column tests whether each group's slope significantly differs from that of the sCAA group.

#### 3.3.2 Memory

Across all CAA groups, memory scores showed a modest overall improvement over time (*F*[1, 121.2] = 5.50, *p* = 0.021; *β* = +0.211 ± 0.069). Baseline memory performance did not differ significantly between groups (*F*[2, 139.1] = 1.53, *p* = 0.220). Model‐based means were −0.547 ± 0.063 for D‐CAA ICH−, −0.689 ± 0.165 for D‐CAA ICH+, and −0.578 ± 0.063 for sCAA, with no significant group differences (largest Δ = 0.14 SD, 95% CI: −0.29 to 0.59, *p* = 0.100). The Time × Group interaction was significant (*F*[2, 126.3] = 4.64, *p* = 0.011), indicating divergent longitudinal trajectories. Post hoc slopes showed a significant improvement in memory for D‐CAA ICH− over time (*β* = +0.260 SD per visit, 95% CI: 0.152 to 0.369, *p* < 0.001 vs 0). The slope in D‐CAA ICH− did not differ from that in sCAA (*p* = 0.79 vs sCAA). D‐CAA ICH+ showed a numerically negative slope (*β* = −0.130 SD per visit, 95% CI: −0.362 to 0.102, *p* = 0.27 vs 0), which did differ significantly from the slope in sCAA (*p* = 0.026 vs sCAA). sCAA showed a small positive slope (*β* = +0.211 SD per visit, 95% CI: −0.003 to 0.425), consistent with relative stability. Thus, the significant Time × Group interaction reflected divergent trajectories, with improvement in D‐CAA ICH− and decline in D‐CAA ICH+. Memory performance in sCAA remained relatively stable over time. Baseline memory strongly predicted follow‐up scores (*β* = 0.854 ± 0.050, *F*[1, 76.5] = 287.7, *p* < 0.001).

#### 3.3.3 Processing speed

Processing speed showed no overall change over time (*F*[1, 184.7] = 0.10, *p* = 0.755; *β* = +0.044 ± 0.055). Baseline performance did not differ significantly between groups (*F*[2, 204.2] = 1.30, *p* = 0.275). Model‐based means were 0.302 ± 0.077 for D‐CAA ICH−, −0.211 ± 0.100 for D‐CAA ICH+, and 0.081 ± 0.076 for sCAA. D‐CAA ICH− showed significantly higher baseline processing speed scores than D‐CAA ICH+ (Δ = 0.51 SD, 95% CI: 0.20 to 0.81, *p* < 0.001), whereas differences with sCAA were non‐significant (ICH− vs sCAA: Δ = 0.22 SD, *p* = 0.132; ICH+ vs sCAA: Δ = 0.29 SD, *p* = 0.067). The Time × Group interaction was significant (*F*[2, 171.9] = 11.44, *p* < 0.001), indicating divergent trajectories across groups. D‐CAA ICH+ showed a significant within‐group decline (*β* = −0.204 SD per visit, 95% CI: −0.313 to −0.094, *p* < 0.001 vs 0), which was also significantly steeper than the slope in sCAA (*p* = 0.001 vs sCAA). D‐CAA ICH− showed a modest improvement (*β* = +0.147 SD per visit, 95% CI: 0.063 to 0.231, *p* = 0.001 vs 0). However, this improvement did not differ significantly from sCAA (*p* = 0.307 vs sCAA). sCAA showed only minimal change over time (*β* = +0.057 SD per visit, 95% CI: −0.040 to 0.154). Thus, the decline in processing speed was specific to D‐CAA ICH+, while other groups remained stable or improved slightly. Neither new sICH (*p* = 0.908) nor its interaction with time (*p* = 0.766) affected outcomes. Baseline processing speed scores strongly predicted follow‐up performance within the same domain (*β* = 0.886 ± 0.036, *F*[1, 180.0] = 619.4, *p* < 0.001).

#### 3.3.4 Executive function

**FIGURE 3:**
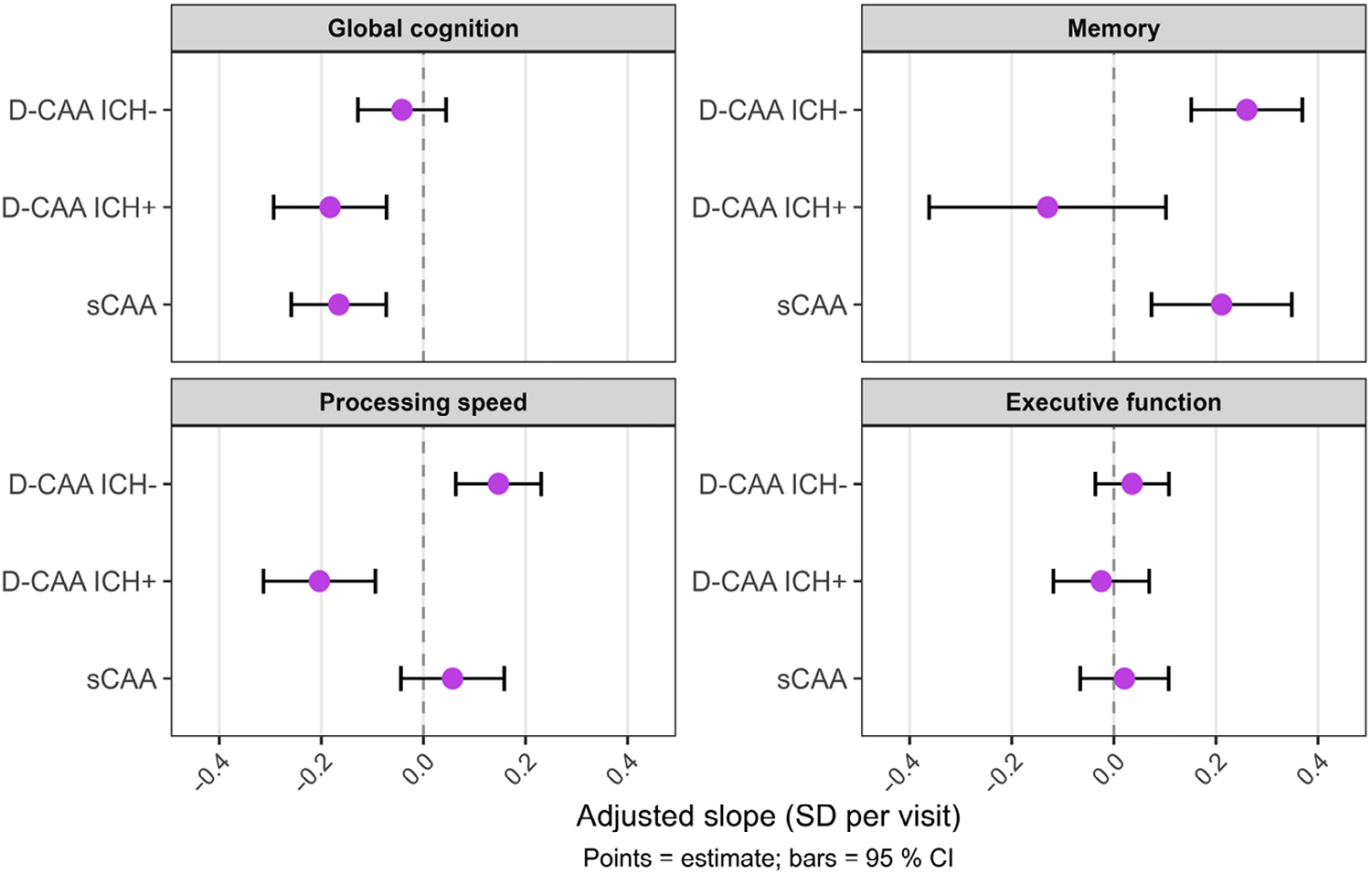
Forest plot.

Executive function remained stable over time across all groups (*F*[1, 192.3] = 0.20, *p* = 0.653; *β* = −0.020 ± 0.044). Baseline performance did not differ significantly between groups (*F*[2, 214.1] = 0.37, *p* = 0.690). Model‐based means were −1.011 ± 0.059 for D‐CAA ICH−, −1.035 ± 0.078 for D‐CAA ICH+, and −1.084 ± 0.059 for sCAA, with no significant pairwise differences (largest Δ = 0.07 SD, *p* = 1.000). The Time × Group interaction was not significant (*F*[2, 191.9] = 0.52, *p* = 0.596), indicating parallel trajectories across all groups. Post hoc slopes confirmed the absence of longitudinal change: D‐CAA ICH− (*β* = +0.036 SD per visit, 95% CI: −0.036 to +0.108), D‐CAA ICH+ (*β* = −0.025, 95% CI: −0.118 to +0.069), and sCAA (*β* = +0.021) all remained stable over time. Baseline executive performance strongly predicted follow‐up scores (*β* = 0.772 ± 0.043, *F*[1, 193.1] = 324.1, *p* < 0.001). Executive function was consistently low at baseline and remained unchanged during follow‐up, with no effect of CAA subtype or hemorrhagic status. Adjusted group differences in longitudinal slopes are summarized in Figure 3.

To complement the analyses of continuous cognitive trajectories, we explored the proportion of participants showing clinically significant cognitive decline, defined as a decrease of ≥1 SD from baseline. Across domains, clinically significant decline occurred in a minority of participants but was generally more frequent in the D‐CAA ICH+ group, particularly for global cognition and processing speed. In contrast, lower proportions of decline were observed in presymptomatic D‐CAA mutation carriers (ICH−) and patients with sCAA. Detailed results are presented in Table S7.

### 3.4 Sensitivity analysis after excluding visits within a year after sICH

Two complementary sensitivity analyses were performed to test the robustness of our findings. First, to verify that our results were not driven by assessments conducted shortly after a sICH, all LMMs were re‐estimated after excluding these visits (Table S3). In total, 46 follow‐up assessments (4.1% of all visits) were removed across participants. For global cognition, the baseline group effect remained non‐significant (main model: *F*[2, 473.9] = 0.63, *p* = 0.533; sensitivity: *F*[2, 432.5] = 0.88, *p* = 0.417]. The previously borderline Time × Group interaction (*F*[2, 426.0] = 3.01, *p* = 0.050) became non‐significant (*F*[2, 395.8) = 2.31, *p* = 0.101). For memory, the baseline group effect remained non‐significant (main: *F*[2, 139.1] = 1.53, *p* = 0.220; sensitivity: *F*[2, 134.6] = 1.34, *p* = 0.265), while the Time × Group interaction persisted (*F*[2, 126.3] = 4.64, *p* = 0.011; sensitivity: *F*[2, 132.8] = 3.87, *p* = 0.023), confirming stable group‐dependent effects. For processing speed, both models yielded consistent results. The baseline group effect remained non‐significant (main model: *F*[2, 204.2] = 1.30, *p* = 0.275; sensitivity: *F*[2, 213.8] = 0.93, *p* = 0.395), and the significant Time × Group interaction persisted (main: *F*[2, 171.9] = 11.44, *p* < 0.001; sensitivity: *F*[2, 177.8] = 10.66, *p* < 0.001). For executive function, results were unchanged, with no main group effect (main: *F*[2, 214.1] = 0.37, *p* = 0.690; sensitivity: *F*[2, 225.2] = 0.66, *p* = 0.518) and no Time × Group interaction (main: *F*[2, 191.9] = 0.52, *p* = 0.596; sensitivity: *F*[2, 197.2] = 0.51, *p* = 0.602).

Together, these analyses confirm that the main longitudinal findings were robust and not driven by short‐term post‐hemorrhagic effects on cognitive functions. The most consistent group‐dependent changes were observed for processing speed and memory, whereas global cognition and executive function remained largely unchanged after exclusion of these visits.

### 3.5 Sensitivity analysis sCAA ICH‐ versus sCAA ICH+

To examine whether a history of sICH influenced longitudinal trajectories in sCAA, we repeated all mixed‐effects models after subdividing the sCAA group into patients without (sCAA ICH−, *n *= 57) and with (sCAA ICH+, *n *= 31) prior sICH. Results were consistent with the main analyses (Table S5). There was a significant Time × Group interaction for global cognition (*F*[3, 434.5] = 3.08, *p* = 0.027), memory (*F*[3, 125.4] = 3.94, *p *= 0.011), and processing speed (*F*[3, 339.8] = 9.40, *p* < 0.001), but not for executive function (*F*[3, 364.7] = 0.57, *p* = 0.630).

Post hoc slopes showed that D‐CAA ICH+ exhibited the steepest decline across domains (global cognition: *β* = −0.17 [95% CI: −0.26 to −0.07]; processing speed: *β* = −0.19 [95% CI: −0.28 to −0.09]). In contrast, D‐CAA ICH− and sCAA ICH− remained largely stable (*β* between −0.04 and +0.13), while sCAA ICH+ showed a modest decline in global cognition (*β* = −0.20 [95% CI: −0.32 to −0.09]) but stable performance in other domains. No subgroup differences were found for executive function, which remained unchanged over time in all groups.

## 4 DISCUSSION

This 5‐year longitudinal study provides novel insights into domain‐specific cognitive trajectories in CAA. Cognitive decline was subtle and stage‐dependent, primarily observed in symptomatic D‐CAA and relative stability in presymptomatic and sporadic cases. Patients with symptomatic D‐CAA (ICH+) showed progressive decline in memory and processing speed, whereas presymptomatic D‐CAA mutation carriers (ICH−) and patients with sCAA remained mostly stable. Processing speed trajectories diverged, with decline in symptomatic D‐CAA and slight improvement in presymptomatic carriers, likely reflecting practice effects. Executive function was impaired across all groups at baseline and remained stable. No clear cognitive decline followed new sICH.

Global cognition declined modestly over time. At each assessment, cognitive performance was standardized relative to age‐, sex‐, and education‐adjusted normative data derived from the Dutch ANDI database. However, because these normative data are based on cross‐sectional reference samples rather than longitudinal follow‐up data, no formal comparison with longitudinal population‐based aging trajectories was performed. Therefore, caution is warranted when interpreting these findings as reflecting disease‐specific rather than age‐related processes.^31^ Similar patterns have been reported in CAA and other types of CSVD.^32^, ^33^ The absence of additional decline after new sICH contrasts with reports of accelerated cognitive deterioration post‐lobar ICH,^34^ likely due to the inclusion of predominantly patients with a CAA ICH phenotype and a limited proportion with a vascular cognitive impairment (VCI)‐like vascular risk profile. Overall, these findings suggest that symptomatic CAA, particularly in individuals with prior ICH, may show gradual, stage‐dependent cognitive deterioration rather than a rapidly progressive trajectory.

Traditionally, CAA‐related cognitive impairment is described as predominantly vascular, with slowed processing speed and executive dysfunction with relatively preserved memory.^16^ Cross‐sectional studies show this differs from that often observed in AD, where episodic memory loss is generally more prominent, although clinical variants exist.^16^, ^19^ Our results refine this concept: Cognition in CAA evolves with disease stage. Subtle memory impairments were detectable in presymptomatic D‐CAA, while progressive memory loss became apparent only once individuals entered the symptomatic stage. This aligns with the biology of hereditary CAA, where the Dutch APP mutation leads to early vascular amyloid deposition that can disrupt neural networks years before ICH occurs.^3^, ^35^ Whether a comparable process exists in sCAA remains uncertain, given that diagnosis typically occurs after symptom onset, limiting characterization of presymptomatic phases.

Neuropathological and imaging data support this interpretation. Severe CAA pathology is associated with dementia independently of plaques or tangles, and amyloid PET shows vascular amyloid years before symptom onset. Fludeoxyglucose (FDG)‐PET studies similarly show hypometabolism predicting future cognitive decline.^36^, ^37^ Together, these findings suggest that a symptomatic hemorrhage may represent a biological tipping point, where cumulative vascular amyloid and microstructural injury exceed the brain's compensatory capacity. In addition, APOE genotype may contribute to heterogeneity in cognitive trajectories and hemorrhagic risk in CAA, particularly through its association with amyloid‐related vascular injury and dementia risk.^38^ Future studies should further investigate the interaction between APOE status, neuroimaging markers, and longitudinal cognitive decline in hereditary and sporadic CAA. However, the present findings do not allow for definitive conclusions regarding the underlying pathophysiological mechanisms. In addition to neurodegenerative processes and overlap with AD, other factors such as vascular inflammation and co‐pathology, particularly concomitant AD pathology or other age‐related neurodegenerative changes, may contribute to the observed cognitive trajectories. These mechanisms likely interact and warrant further investigation using multimodal biomarkers.

Interestingly, no additional cognitive decline was observed following new sICH. Acute ICH may not be the primary driver of 5‐year cognitive deterioration in this cohort. We characterized ICH location (predominantly lobar), but more detailed characteristics such as hemorrhage size or volume were not consistently available and could therefore not be incorporated. Such factors may influence cognitive outcomes and should be considered in future studies. Instead, the gradual accumulation of amyloid‐related vascular injury may contribute to progressive decline, particularly in symptomatic D‐CAA.

In sCAA, greater clinical and pathological heterogeneity may contribute to variable cognitive trajectories. Notably, the sCAA group in this study showed a relatively high hemorrhagic marker burden, as reflected by the high number of CMBs and prevalence of cortical superficial siderosis. This likely reflects the tertiary referral setting and may limit the generalizability of our findings to patients with milder or earlier‐stage sCAA. Moreover, this high disease burden may have influenced the observed cognitive trajectories within the sCAA group. While an individual ICH may cause stepwise worsening,^34^ our results imply that the ongoing accumulation of amyloid‐related vascular injury is likely an important contributing factor to progressive cognitive decline. Subgroup analyses by ICH status showed divergent trajectories, but these findings should be interpreted cautiously due to increased heterogeneity and reduced statistical power. These results support modeling sCAA as a single group in the primary analyses.

Processing speed decline was most pronounced in patients with symptomatic D‐CAA, consistent with earlier findings in our cohort linking CAA severity to slowed information processing.^4^, ^20^ Progressive white matter disconnection and cortical–subcortical network disruption likely underlie this gradual slowing.^39^, ^40^, ^41^ In those with prior ICH, residual structural damage may contribute to chronic vascular injury. By contrast, presymptomatic D‐CAA carriers showed mild improvement across domains, including processing speed and memory, most plausibly reflecting practice effects due to repeated administration of identical test versions^42^, ^43^ or ceiling effects in individuals with relatively high baseline performance. Clinically, slowing of processing speed may serve as a marker of disease progression, whereas apparent improvement or stability in early stages warrants careful interpretation, as both may precede measurable cognitive decline.

Executive dysfunction was evident across all CAA groups at baseline and remained stable. Importantly, most mean baseline *Z*‐scores did not indicate overt cognitive impairment at the group level and should be interpreted as reflecting subtle between‐group differences in performance rather than clear‐cut impairment. However, this finding should be interpreted cautiously, as the relatively low executive scores were partly driven by low scores on the Digit Span backward task. This pattern suggests that the low executive composite scores may partly reflect task‐specific characteristics and, to some extent, the normative scaling of this measure, rather than disease‐specific executive impairment. This pattern is consistent with chronic disconnection from small‐vessel disease, where further decline may be limited once a critical lesion burden is reached.^16^ Such sustained but non‐progressive executive deficits are typical of VCI, though larger or longer‐term studies might still reveal subtle decline. In contrast to reports of post‐ICH dementia,^34^ our results likely reflect differences in disease stage and outcome definition (domain‐specific changes vs new‐onset dementia diagnosis).

The strengths of this study include its well‐characterized hereditary cohort, prospective longitudinal follow‐up, and standardized annual neuropsychological assessments. This allowed precise modeling of within‐person cognitive change and between‐group differences across disease stages.

We acknowledge several limitations. First, although 5 years is relatively long, CAA‐related cognitive decline likely unfolds over decades. Second, because of the annual assessment interval and the selective inclusion of patients able to return for follow‐up, the study was unable to detect acute or early post‐ICH cognitive effects of new sICH. Moreover, the study population likely represents a select subgroup of patients with sufficient functional and cognitive capacity to participate in repeated assessments. Patients with more severe post‐ICH deficits, such as aphasia, neglect, or severe disability, may have been underrepresented, which could lead to an underestimation of the impact of ICH on cognitive decline and limit the generalizability of our findings. Third, repeated test exposure to the same cognitive test versions may have introduced practice effects, particularly in memory and processing speed, as parallel test forms were not available. Additionally, during a limited period following COVID‐19‐related interruptions, only the MoCA was administered instead of the full neuropsychological test battery to accommodate a high number of rescheduled follow‐up visits. Although these assessments were conducted in person, this temporary protocol adaptation reduced the availability of complete cognitive data. Fourth, outcome missingness was substantial for several cognitive domains and arose from multiple sources, including protocol amendments, COVID‐19‐related restrictions, incomplete follow‐up visits, and early termination of neuropsychological assessments. Although LMMs can accommodate unbalanced data, we cannot exclude the possibility that missingness related to clinical deterioration may have biased estimates of longitudinal cognitive change. Importantly, despite this missingness, we still observed consistent patterns of cognitive trajectories across groups, suggesting that the main findings are robust. Finally, heterogeneity in CAA severity and ICH characteristics was not fully captured, which may influence cognitive outcomes. Grouping patients by hemorrhagic status simplifies classification, but it cannot fully capture this complexity. However, although we included a descriptive characterization of ICH location at baseline, more detailed characteristics such as hemorrhage size or volume were not systematically incorporated into the analyses, which limits the interpretation of ICH‐related cognitive effects. Future studies may further investigate how specific neuroimaging markers of CAA relate to longitudinal cognitive decline and domain‐specific cognitive trajectories.

In summary, CAA may contribute to slow but progressive cognitive decline, with the pattern and pace depending on disease stage and clinical presentation. Memory and processing speed deteriorate mainly in symptomatic stages, whereas executive deficits appear early and remain stable. These findings underscore the importance of longitudinal cognitive monitoring in CAA and highlight that subtle slowing or emerging memory decline may signal advancing small vessel pathology.

## CONFLICT OF INTEREST STATEMENT

Rosemarie van Dort, Reinier G.J. van der Zwet, and Ellen P. Hart are funded by the TRACK D‐CAA consortium, consisting of Biogen, Alnylam, the Dutch CAA foundation, Vereniging HCHWA‐D, and researchers from Leiden, Boston, and Perth. Manon R. Schipper receives financial support for post‐processing of clinical trial data by Alnylam, Clario, and Advanced Clinical LLC, and has received independent support from the TRACK D‐CAA consortium. Hamid R. Sohrabi, Vandhana Easwaran, Samantha L. Gardener, Kevin Taddei, and Ralph N. Martins report no conflict of interest directly related to this work. Hamid R. Sohrabi, Kevin Taddei, and Ralph N. Martins have had or are receiving research support from Alzheimer's Research Australia as well as Pharmaceutical and Nutraceutical companies including Alector, Alnylam, CWEKPTYLTD, WA, Australia, and Biogen. Marieke J.H. Wermer reports independent support from the Dutch Heart Foundation (Dekker grant 2016T086). Matthias J.P. van Osch reports support by a NWO‐Human Measurement Models 2.0 grant (18969) as well as support from the Dutch Research Council (NWO), a grant from the Leducq Foundation, the Leducq Foundation for Cardiovascular Research (23CVD03), and the Dutch Brain Foundation. Matthias J.P. van Osch, Steven M. Greenberg, and Ellis S. van Etten are unpaid members of the clinical trial steering committee of the cAPPricorn trial of Alnylam. Ellis S. van Etten further reports support from Alzheimer Nederland (Project ID: 642991) and the Dutch Research Council (NWO) (Veni; ID: 09150162410011). The other authors report no conflict of interest. Author disclosures are available in the Supporting Information.

## CONSENT STATEMENT

All participants provided informed consent before their enrollment.

## Supporting information



Supporting Information

Supporting Information
