# Supplementary material for: Cognitive decline in Dutch‐type hereditary and sporadic cerebral amyloid angiopathy: a 5‐year follow‐up study
Source: Alzheimers Dement. 2026 Jun 26;22(7):e71629. doi: 10.1002/alz.71629 (PMC13307620; doi:10.1002/alz.71629)
Supplement: Supplementary file 1 — Supporting Information [file ALZ-22-e71629-s001.docx]

**SUPPLEMENTARY TABLES**

**Table S1. Number of completed neuropsychological assessments**

| **Group** | **N (baseline)** | **Baseline** | **1 FU** | **2 FUs** | **≥ 3 FUs** | **Total n** |
| --- | --- | --- | --- | --- | --- | --- |
| **D-CAA ICH–** | 59 | 30 | 80 | 85 | 100 | **295** |
| **D-CAA ICH+** | 34 | 20 | 15 | 15 | 120 | **170** |
| **sCAA** | 88 | 135 | 95 | 85 | 125 | **440** |

*Note*. N represents the number of participants per group at baseline. Values per follow-up year represent the number of completed neuropsychological assessments. Values represent the total number of completed neuropsychological assessments (visits) available per CAA group at each follow-up year. Counts therefore reflect visits rather than unique participants, as individuals may contribute multiple follow-up assessments.

**Table S2. Availability of cognitive outcome measures across groups**

| **Group** | **Cognitive domain** | **Available observations** | **Missing observations** | **Missing (%)** |
| --- | --- | --- | --- | --- |
| **D-CAA ICH−** | Global cognition | 178 | 176 | 49.7 |
|  | Memory | 120 | 234 | 66.1 |
|  | Processing speed | 150 | 204 | 57.6 |
|  | Executive function | 152 | 202 | 57.1 |
| **D-CAA ICH+** | Global cognition | 96 | 79 | 45.1 |
|  | Memory | 55 | 120 | 68.6 |
|  | Processing speed | 80 | 95 | 54.3 |
|  | Executive function | 80 | 95 | 54.3 |
| **sCAA** | Global cognition | 203 | 308 | 60.3 |
|  | Memory | 111 | 400 | 78.3 |
|  | Processing speed | 153 | 358 | 70.1 |
|  | Executive function | 156 | 355 | 69.5 |

*Note*. Outcome availability differed across cognitive domains and visits. Missing outcome data resulted from several sources, including protocol amendments (e.g., the RAVLT was not included before June 2019), COVID-19–related restrictions during which only the MoCA was administered, incomplete follow-up visits, and early termination of neuropsychological assessments. Longitudinal analyses were performed using linear mixed-effects models, which allow inclusion of all available repeated observations. Abbreviations: CAA = cerebral amyloid angiopathy; ICH = intracerebral hemorrhage.

**Table S3. Sensitivity analysis after excluding visits within one year after sICH**

| \| **Cognitive domain** \| **Effect tested** \| **Main analysis** \| **Sensitivity analysis** \| **Interpretation** \| \| --- \| --- \| --- \| --- \| --- \| \| **Global cognition** \| Group (baseline) \| *F*(2, 473.9) = 0.630, *p*= 0.533 \| *F*(2, 432.4)=0.88, *p*= 0.417 \| Remained non-significant \| \|  \| Time × Group \| *F*(2, 426.0) = 3.01, *p*= 0.050 \| *F*(2, 395.8)=2.31, *p*= 0.101 \| Interaction no longer significant \| \| **Memory** \| Group (baseline) \| *F*(2, 139.1) = 1.53, *p*= 0.220 \| *F*(2, 134.6)=1.34, *p*= 0.265 \| Remained non-significant \| \|  \| Time × Group \| *F*(2, 126.3) = 4.64, *p*= 0.011 \| *F*(2, 132.8)=3.87, *p*= 0.023 \| Remained significant \| \| **Processing speed** \| Group (baseline) \| *F*(2, 204.2) = 1.30, *p*= 0.275 \| *F*(2, 213.8)=0.93, *p*= 0.395 \| Remained non-significant \| \|  \| Time × Group \| *F*(2, 171.9) = 11.44, *p*< 0.001 \| *F*(2, 178)=10.66, *p*< 0.001 \| Remained significant \| \| **Executive function** \| Group (baseline) \| *F*(2, 214.1) = 0.37, *p*= 0.690 \| *F*(2, 225.2)=0.66, *p*= 0.518 \| Remained non-significant \| \|  \| Time × Group \| *F*(2, 191.9) = 0.52, *p*= 0.596 \| *F*(2, 197.2)=0.51, *p*= 0.602 \| Remained non-significant \| |
| --- | --- | --- | --- | --- | --- | --- | --- | --- | --- | --- | --- | --- | --- | --- | --- | --- | --- | --- | --- | --- | --- | --- | --- | --- | --- | --- | --- | --- | --- | --- | --- | --- | --- | --- | --- | --- | --- | --- | --- | --- | --- | --- | --- | --- | --- |

*Note.* Models included fixed effects for Time × Group with random intercepts for participants. All estimates are adjusted Z-scores per cognitive domain. Results reflect sensitivity analyses excluding visits conducted within one year after symptomatic intracerebral hemorrhage.

**Table S4. Baseline characteristics sCAA ICH− vs ICH+**

|  | **sCAA ICH-** | **sCAA ICH+** |
| --- | --- | --- |
| n | 57 | 31 |
| Age, mean, y (SD) | 70.4 (6.5) | 70.6 (7.2) |
| Women, n (%) | 24 (44.4%) | 11 (35.5%) |
| First CAA-related complaints, n (%) |  |  |
| ICH | - | - |
| (Subjective) cognitive decline | 23 (74.2%) | 18 (64.3%) |
| Transient focal neurological episodes | 10 (34.5%) | 7 (28.0%) |
| Seizures | 0 (0.0%) | 0 (0.0%) |
| Convexity subarachnoid hemorrhage | 0 (0.0%) | 0 (0.0%) |
| Other | 0 (0.0%) | 0 (0.0%) |
| None/Genetic testing/Research | 0 (0.0%) | 0 (0.0%) |
| History of symptomatic ICH, n (%)* | - | 31 (100.0%) |
| Number of symptomatic ICH, median [IQR] | - | 1 [1-2] |
| History of depression, n (%) | 11 (20.8%) | 6 (17.6%) |
| History of subjective personality change †, n (%) | 31 (58.5%) | 22 (64.7%) |
| History of apathy, n (%) | 6 (11.3%) | 8 (25.8%) |
| Education, y (SD) | 13.5 (3.5) | 14.7 (4.2) |
| Education level, n (%) |  |  |
| High | 7 (18.4%) | 1 (3.2%) |
| Average | 6 (15.8%) | 2 (6.5%) |
| Low | 8 (21.1%) | 2 (6.5%) |
| Hypertension, n (%) | 26 (50.0%) | 18 (52.9%) |
| Hypercholesterolemia, n (%) | 22 (44.9%) | 15 (45.5%) |
| Diabetes mellitus type II, n (%) | 5 (9.4%) | 2 (6.5%) |
| Smoking (ever), n (%) | 38 (71.7%) | 22 (64.7%) |
| Alcohol use (ever), n (%) | 49 (92.5%) | 27 (79.4%) |

*Note.* CSO-EPVS, centrum semiovale enlarged perivascular spaces; D-CAA, Dutch-type cerebral amyloid angiopathy; DWMH, deep white matter hyperintensities; sCAA, sporadic cerebral amyloid angiopathy; ICH, intracerebral hemorrhage. Education level data were missing for 40 participants (16 sCAA ICH– and 27 sCAA ICH+); percentages are based on available data.
* Recorded only for patients with history of symptomatic ICH. † Collected as personal experience of change in character during the most recent years

**Table S5. Sensitivity analysis sCAA ICH− vs ICH+**

| \| **Cognitive domain** \| **Effect tested** \| **Main analysis** \| **Sensitivity analysis  (sCAA split)** \| **Interpretation** \| \| --- \| --- \| --- \| --- \| --- \| \| **Global cognition** \| Group (baseline) \| *F*(2, 473.9)= 0.63, *p*= 0.533 \| *F*(3, 367.1)= 1.69, *p*= 0.169 \| Remained non-significant \| \|  \| Time × Group \| *F*(2, 426.0)= 3.01, *p*= 0.050 \| *F*(3, 434.5)= 3.08, *p*= 0.027* \| Remained significant \| \| **Memory** \| Group (baseline) \| *F*(2, 139.1)= 1.53, *p*= 0.220 \| *F*(3, 125.4)= 3.94, *p*= 0.011* \| Becomes significant \| \|  \| Time × Group \| *F*(2, 126.3)= 4.64, *p*= 0.011 \| *F*(3, 125.4)= 3.94, *p*= 0.011 \| Remained significant \| \| **Processing speed** \| Group (baseline) \| *F*(2, 204.2)= 1.30, *p*= 0.275 \| *F*(3, 339.8)= 9.40, *p*< 0.001* \| Becomes significant \| \|  \| Time × Group \| *F*(2, 171.9)= 11.44, *p*< 0.001 \| *F*(3, 339.8)= 9.40, *p*< 0.001 \| Remained significant \| \| **Executive function** \| Group (baseline) \| \| *F*(2, 214.1)= 0.37, *p*= 0.690 \| \| --- \| \| *F*(3, 364.7)= 0.57, *p*= 0.630 \| Remained non-significant \| \|  \| Time × Group \| *F*(2, 191.9)= 0.52, *p*= 0.596 \| *F*(3, 364.7)= 0.57, *p*= 0.630 \| Remained non-significant \| |
| --- | --- | --- | --- | --- | --- | --- | --- | --- | --- | --- | --- | --- | --- | --- | --- | --- | --- | --- | --- | --- | --- | --- | --- | --- | --- | --- | --- | --- | --- | --- | --- | --- | --- | --- | --- | --- | --- | --- | --- | --- | --- | --- | --- | --- | --- | --- |

*Note.* Models included fixed effects for Time × Group with random intercepts for participants. All estimates are adjusted Z-scores per cognitive domain. This sensitivity analysis subdivided the sCAA group into participants without (ICH–) and with (ICH+) a history of symptomatic intracerebral hemorrhage.

**Table S6. Prevalence of domain-specific cognitive dysfunction at baseline**

| **Domain** | **D-CAA ICH-** | **D-CAA ICH+** | **sCAA** |
| --- | --- | --- | --- |
| **Global, Z ≤ -1** | 5/59 (8.5%) | 6/28 (21.4%) | 20/83 (24.1%) |
| **Global ≤ -2** | 0/59 (0%) | 0/28 (0%) | 12/83 (14.5%) |
| **Memory ≤ -1** | 9/32 (28.1%) | 1/5 (20%) | 20/45 (44.4%) |
| **Memory ≤ -2** | 2/32 (6.2%) | 0/5 (0%) | 5/45 (11.1%) |
| **Processing speed ≤ -1** | 5/58 (8.6%) | 2/27 (7.4%) | 22/77 (28.6%) |
| **Processing speed ≤ -2** | 2/58 (3.4%) | 1/27 (3.7%) | 10/77 (13%) |
| **Executive function ≤ -1** | 29/59 (49.2%) | 15/27 (55.6%) | 50/78 (51.3%) |
| **Executive function ≤ -2** | 7/59 (11.9%) | 2/27 (7.4%) | 15/78 (19.2%) |

*Note.* Data are presented as n/N (%). Cognitive dysfunction was defined as a domain-specific Z-score ≤ -1 SD relative to normative data. As a sensitivity analysis, a more stringent cut-off of Z ≤ -2 SD was applied. Due to protocol-related missingness, the number of participants with available data differed by domain.

**Table S7. Frequency of clinically significant cognitive decline (≥1 SD from baseline)**

| **Group** | **Cognitive domain** | **Participants with follow-up (n)** | **Participants with ≥1 SD decline (n)** | **Decline (%)** |
| --- | --- | --- | --- | --- |
| **D-CAA ICH−** | Global cognition | 52 | 10 | 19.2 |
| **D-CAA ICH+** | Global cognition | 24 | 12 | 50.0 |
| **sCAA** | Global cognition | 55 | 24 | 43.6 |
| **D-CAA ICH−** | Memory | 23 | 2 | 8.7 |
| **D-CAA ICH+** | Memory | 3 | 0 | 0 |
| **sCAA** | Memory | 22 | 0 | 0 |
| **D-CAA ICH−** | Processing speed | 46 | 5 | 10.9 |
| **D-CAA ICH+** | Processing speed | 23 | 12 | 52.2 |
| **sCAA** | Processing speed | 49 | 9 | 18.4 |
| **D-CAA ICH−** | Executive function | 47 | 3 | 6.4 |
| **D-CAA ICH+** | Executive function | 23 | 3 | 13.0 |
| **sCAA** | Executive function | 50 | 9 | 18.0 |

*Note*. Clinically significant cognitive decline was defined as a decrease of ≥1 standard deviation from the baseline Z-score in the respective cognitive domain during follow-up. Percentages represent the proportion of participants with available follow-up data for that domain. Participants with follow-up represent individuals with at least one follow-up assessment for the respective cognitive domain.

**
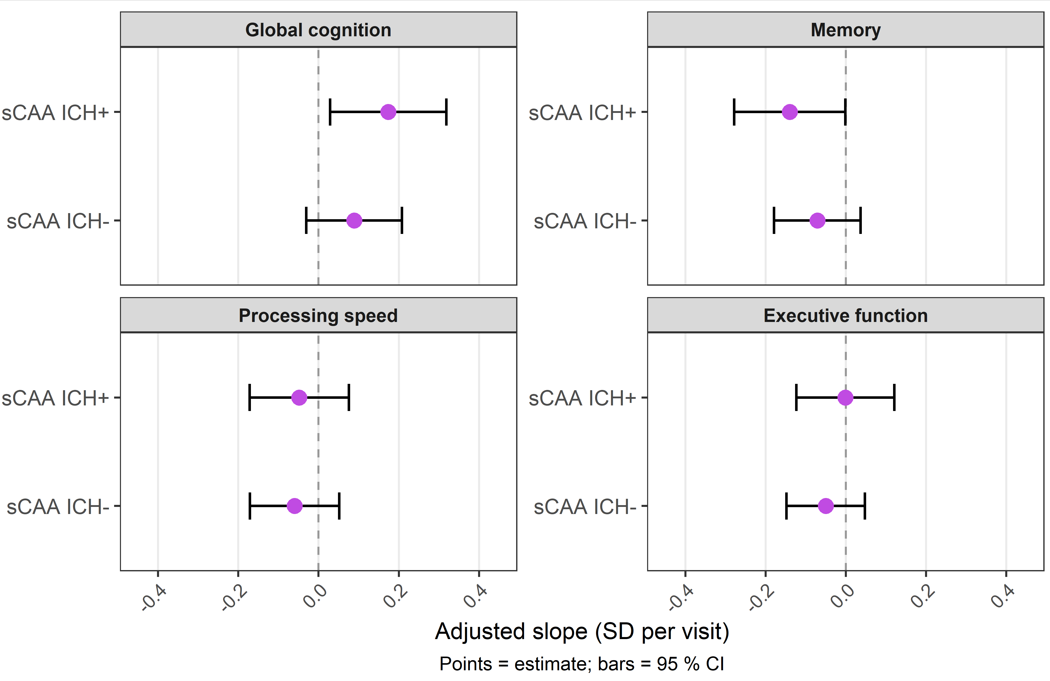
SUPPLEMENTARY FIGURES**

**Figure S1. Sensitivity analysis within sCAA (ICH- vs ICH+)**
